# Applications of genotyping by sequencing in aquaculture breeding and genetics

**DOI:** 10.1111/raq.12193

**Published:** 2017-02-04

**Authors:** Diego Robledo, Christos Palaiokostas, Luca Bargelloni, Paulino Martínez, Ross Houston

**Affiliations:** ^1^ The Roslin Institute and Royal (Dick) School of Veterinary Studies University of Edinburgh Midlothian UK; ^2^ Department of Comparative Biomedicine and Food Science University of Padova Legnaro Padova Italy; ^3^ Department of Zoology Genetics and Physical Anthropology Faculty of Veterinary University of Santiago de Compostela Lugo Spain

**Keywords:** aquaculture, genotyping, next‐generation sequencing, restriction‐site associated DNA, selective breeding, single nucleotide polymorphism

## Abstract

Selective breeding is increasingly recognized as a key component of sustainable production of aquaculture species. The uptake of genomic technology in aquaculture breeding has traditionally lagged behind terrestrial farmed animals. However, the rapid development and application of sequencing technologies has allowed aquaculture to narrow the gap, leading to substantial genomic resources for all major aquaculture species. While high‐density single‐nucleotide polymorphism (SNP) arrays for some species have been developed recently, direct genotyping by sequencing (GBS) techniques have underpinned many of the advances in aquaculture genetics and breeding to date. In particular, restriction‐site associated DNA sequencing (RAD‐Seq) and subsequent variations have been extensively applied to generate population‐level SNP genotype data. These GBS techniques are not dependent on prior genomic information such as a reference genome assembly for the species of interest. As such, they have been widely utilized by researchers and companies focussing on nonmodel aquaculture species with relatively small research communities. Applications of RAD‐Seq techniques have included generation of genetic linkage maps, performing genome‐wide association studies, improvements of reference genome assemblies and, more recently, genomic selection for traits of interest to aquaculture like growth, sex determination or disease resistance. In this review, we briefly discuss the history of GBS, the nuances of the various GBS techniques, bioinformatics approaches and application of these techniques to various aquaculture species.

## Background

Despite the critical role for aquaculture in global food security, the vast majority of world fish and shellfish production is based on stocks without advanced selective breeding programmes (Gjedrem *et al*. 2012; Janssen *et al*. 2016). Aquaculture breeding schemes tend to lag behind their terrestrial livestock counterparts in terms of the uptake of genomic technologies, and for many aquaculture species, molecular genetic tools are only applied for pedigree reconstruction (Chavanne *et al*. 2016). In comparison, most modern breeding programmes in livestock are now underpinned by genomic selection (GS, Meuwissen *et al*. 2001), the benefits of which are well‐illustrated in dairy cattle (Hayes *et al*. 2009). GS typically requires genome‐wide genetic marker data for a large number of individual animals. Up until a few years ago, obtaining genetic markers was costly and laborious; hence, large numbers of markers were only available for a handful of well‐studied species. However, the recent advances in next‐generation sequencing (NGS) have greatly reduced the cost of nucleic acid sequencing, and therefore also genetic marker discovery. This has opened the door for rapid generation of genome‐wide genetic marker datasets, either via generation and application of SNP arrays, or directly via genotyping by sequencing (GBS) techniques (Davey *et al*. 2011). GBS techniques have revolutionized the field of evolutionary genomics (reviewed in Andrews *et al*. 2016) and have also led to several advances in genetics and breeding of aquaculture species, the subject of this review.

Due to the high fecundity of aquaculture species, the majority of breeding programmes are based on collection of trait data on close relatives (e.g. full siblings) of the selection candidates, particularly where the trait of interest cannot be measured on the candidates themselves (e.g. fillet quality, disease resistance). Without genetic markers, this set‐up enables family selection, whereby family‐level estimated breeding values (EBVs) for selection candidates are calculated using the data collected on the relatives. However, to utilize the within‐family genetic variation in these traits, genetic markers are necessary to distinguish between selection candidates. Implementation of markers in breeding can broadly be split into two categories; marker‐assisted selection (MAS) and GS. MAS is based on the use of targeted markers linked to major quantitative traits loci (QTL) affecting the trait, and one of the first examples in aquaculture was host resistance to infectious pancreatic necrosis virus (IPNV) in Atlantic salmon (*Salmo salar*, Houston *et al*. 2008; Moen *et al*. 2009). For traits with a polygenic architecture, GS is a more appropriate approach, whereby the relatives of the selection candidates become the ‘training’ population with genotypes and phenotypes, and those data are used to calculate genomic breeding values (GEBVs) for selection candidates with genotype data only. This application of genomic selection in aquaculture breeding is at a formative stage, and most examples to date have focussed on improved breeding for resistance to infectious diseases (e.g. Ødegård *et al*. 2014; Tsai *et al*. 2015, 2016b; Vallejo *et al*. 2016; Dou *et al*. 2016; Palaiokostas *et al*. 2016). The majority of high‐resolution genetic studies in aquaculture species, and applications of genomic selection, have been underpinned by GBS techniques, either by directly providing genotype data or by discovering markers for the design of SNP arrays, which are currently only available for a handful of aquaculture species (e.g. Atlantic salmon, Houston *et al*. 2014; Yáñez *et al*. 2016; Pacific oyster, *Crassostrea gigas*, and European flat oyster, *Ostrea edulis*, Lapègue *et al*. 2014; channel catfish, *Ictalurus punctatus*, Liu *et al*. 2014; common carp, *Cyprinus carpio*, Xu *et al*. 2014a; rainbow trout, *Oncorhynchus mykiss*, Palti *et al*. 2015a).

The most common GBS techniques involve library preparation steps that result in deep sequence data at a repeatable subset of sites dispersed throughout the genome, typically using one or two restriction enzymes (RE), although also new GBS techniques based on targeted sequencing have been recently developed (i.e. GT‐Seq, discussed below). The reason behind this genome complexity reduction is that high‐coverage sequencing of a typical aquaculture species’ genome with enough depth to confidently call genotypes is still prohibitively expensive for the number of animals required for high‐resolution genetic studies and breeding programme applications. Genome complexity reduction via RE is fast and inexpensive. Indeed, RE‐based techniques have been commonplace in genotyping for many years, with RFLP and AFLP being widely applied to generate genotyping assays for limited numbers of genetic markers. The marriage of these ideas with NGS has enabled a major breakthrough for genetic studies of complex traits in nonmodel organisms, and their application to improve aquaculture production.

## RAD sequencing

Restriction‐site associated DNA sequencing (RAD sequencing or RAD‐Seq) covers a range of GBS techniques which combine the use of genome complexity reduction with REs and the high sequencing output of NGS technologies. RAD‐Seq was first described by Baird *et al*. (2008), following on from a similar idea based on microarrays (Miller *et al*. 2007). Some of the main reasons for its instant success are that RAD‐Seq does not require any prior genomic knowledge, it allows generation of population‐specific genotype data (i.e. no ascertainment bias) and it offers flexibility in terms of desired marker density across the genome. The use of different REs or innovative modifications to the base technique allows a high level of control over the number of markers obtained for a specific study. RAD‐Seq and similar techniques are also amenable tools for aquaculture breeding, where genetic markers have typically been used in family assignment and pedigree reconstruction (Vandeputte & Haffray 2014). Mass spawning species are common in aquaculture, where mixed rearing and unknown parental contribution necessitate the use of genotyping for family‐based breeding. RAD‐Seq potentially facilitates a single experiment whereby pedigrees are reconstructed, genetic diversity is quantified, QTL can be mapped and genomic breeding values calculated (Palaiokostas *et al*. 2016). Since the original RAD‐Seq paper by Baird *et al*. (2008), several variants of this methodology have been described. Three of them have been extensively used in aquaculture genetics research: the original RAD‐Seq (Baird *et al*. 2008), 2b‐RAD (Wang *et al*. 2012) and ddRAD (Peterson *et al*. 2012). Other RAD‐based techniques like ezRAD (Toonen *et al*. 2013) or SLAF‐seq (Sun *et al*. 2013) introduced minor modifications, which do not confer a major advantage for aquaculture applications. All available RAD‐based techniques have been recently reviewed in depth elsewhere (Andrews *et al*. 2016); therefore, here we have focused on those most relevant in aquaculture breeding. The main features of original RAD‐Seq, 2b‐RAD and ddRAD are shown in Table 1, and they are briefly described below.

**Table 1:** Summary of the different genotyping by sequencing (GBS) techniques

Technique	Key features	Advantages	Disadvantages
RAD‐Seq	Digestion with one RE	Paired‐end contigs PCR duplicate removal	Complex library preparation
2bRAD	Digestion with type IIB REs	No size‐selection step High reproducibility Easy library preparation Strand bias detection	Short fragments Removal of PCR duplicates not possible
ddRAD	Digestion with two different REs	Can multiplex many samples Easy library preparation Flexibility over SNP density	Repeatability dependent on size‐selection step

### Original RAD‐Seq

In original RAD‐Seq (Baird *et al*. 2008), genomic DNA samples from several animals are individually digested with a RE of choice. The digested DNA is then randomly sheared and pooled after ligation of adaptors with nucleotide barcodes for unique identification of each sample. The resulting restriction fragments are selected for suitable size range (i.e. for Illumina sequencing, typically 300–600 bp), and after a subsequent polymerase chain reaction (PCR) step, the fragments are sequenced. The result is high‐coverage sequence data for flanking regions of the RE cut sites, which are typically dispersed quite evenly throughout the genome. As such, a genome‐wide genetic marker dataset can be produced across a population of individuals at a fraction of the cost of whole genome resequencing. Illumina sequencing of short fragments either involves sequencing one (one read, single end) or both (two reads, paired end) ends of each fragment and currently gives reads of up to 300 bp in length. Each flanking sequence of the RE cut site is referred to as a RAD locus (or RAD‐tag), and the high coverage of RAD tags facilitates simultaneous SNP detection and genotyping. The number of RAD tags, and therefore SNPs, generated in the experiment is tuneable via the choice of rarer or more frequent cutting RE. The most commonly used enzyme to date is SbfI which has an eight base recognition site and therefore cuts relatively infrequently throughout the genome. Online tools are available to guide the choice of the most appropriate RE according to the requirements and budget of the study (Lepais & Weir 2014). In addition to sequencing and genotyping individuals, the approach is also amenable to genotyping pooled populations for bulk‐segregant analysis (Baird *et al*. 2008; Hohenlohe *et al*. 2010). One of the main drawbacks of the original technique is that shearing by sonication is random and variable, potentially hindering the efficiency and the reproducibility of RAD‐Seq (Davey *et al*. 2013). However, this random shearing step can also be a benefit, as the variable size of the genomic fragments anchored at the RE cut site facilitates the assembly of a contig based on the paired‐end reads. This augments annotation of the RAD loci when there is no reference genome available, and also the design of specific primers for re‐genotyping of targeted SNPs. In addition, the paired‐end data from RAD‐Seq allow identification and removal of putative PCR duplicates (reads originated from the same original DNA fragment, therefore presenting identical sequences), which can hinder analysis and interpretation of Illumina sequencing data (Schweyen *et al*. 2014). While there are several sources of potential bias and error in RAD‐Seq techniques (see review by Andrews *et al*. 2016), several theoretical and empirical studies have demonstrated that RAD‐Seq does render reproducible genotyping data across different laboratories, populations and even species (e.g. DaCosta & Sorenson 2014; Gonen *et al*. 2015).

### 2b‐RAD

The first major modification of the original RAD technique was termed 2b‐RAD (Wang *et al*. 2012). The main innovation in 2b‐RAD is the use of type IIB REs, which share the feature of cutting the genomic DNA at both sides of the recognition site at a fixed distance, resulting in protruding noncohesive ends. The result is short genomic DNA fragments of identical size at each IIB RE site in the genome. Library construction in the 2b‐RAD protocol is simple. Following DNA digestion, adaptors are ligated to the fragments, and specific barcodes are added to each sample through PCR amplification using degenerated linkers. Samples are then pooled and sequenced typically using Illumina technology, but allowing for runs of shorter read length due to the smaller size of the fragments in comparison to original RAD (2b‐RAD fragments are 33–36 bp). The use of type IIB REs theoretically facilitates the sampling and sequencing of identical sites across individuals, circumventing the potential bias of RAD‐Seq caused by the random shearing step. It also avoids the time‐consuming and potentially error‐prone size‐selection step, which characterizes the majority of other RAD methods. Additionally, 2b‐RAD is currently the only member of the RAD family that allows removal of loci exhibiting strand bias (Puritz *et al*. 2014a). The possibility to produce individually barcoded libraries allows targeted adjustment before pooling to obtain more equal representation of individual samples. The main caveat of this method is that it produces short sequencing reads (33–36 bp), which are less amenable for alignment to reference genome assemblies, and hinders follow‐up applications such as the design of individual SNP assays (due to lack of SNP flanking sequence). However, this is not an issue if a draft genome sequence is available for the species, as is becoming the case in many aquaculture fish species.

### ddRAD

Peterson *et al*. (2012) developed a new RAD‐Seq platform using a double digestion of genomic DNA with two REs (ddRAD), thus eliminating the shearing step of original RAD. The ddRAD protocol is more flexible than RAD‐Seq or 2b‐RAD in terms of targeted marker density; the number of fragments and SNPs can be readily tailored by combining different RE pairs. Due to the typical use of a rare and a common cutting enzyme, ddRAD results in fewer sequenced sites than RAD‐Seq, facilitating higher sequence coverage and/or more individuals multiplexed within a single sequencing lane. Higher multiplexing is possible due to combinational multiplex indexing, whereby a first barcode is introduced in the ligation step and a second during the PCR. Therefore, a larger number of samples can potentially be sequenced in a single lane than with the other RAD techniques. Compared to the RAD‐Seq protocol, the workflow of preparation of ddRAD libraries is simpler, quicker and also substantially cheaper. However, the workflow is still more complex than the 2b‐RAD protocol and requires a size‐selection step. To ensure repeatability of sampled ddRAD loci across samples and libraries, consistency of size selection is paramount (Andrews *et al*. 2016). A simplified variation of the initial ddRAD protocol, where both P1 and P2 adaptors with individual barcodes are ligated prior to size selection (Palaiokostas *et al*. 2015a), further reduces hands‐on time for library preparation.

## RAD bioinformatic analyses

The advent of NGS posed important challenges in terms of data storage, transfer and analysis, which necessitated the development of specialized hardware and software. Consequently, the improvement of NGS‐based sequencing platforms occurred in tandem with continuous development and improvement of suitable bioinformatics tools to analyse the large datasets. A wealth of software is available for analysing data originating from the RAD family of techniques. In the current review, a general framework for data analysis will be described, rather than attempting to provide a comprehensive overview of all available tools. Accordingly, the most popular, straightforward to use and regularly updated of the available tools are highlighted in terms of a suggested order of usage that might form a complete RAD analysis pipeline.

### Experimental design and simulation

Sequencing and library construction typically account for the bulk of the cost of any experiment utilizing NGS. This leads to a balancing exercise, whereby researchers strive to include as many samples as possible per sequencing lane (multiplexing), without compromising the read coverage required for accurate SNP genotype calling. Therefore, two key variables for a RAD experiment are the choice of the RE (affecting how many sites are sequenced), and the desired read coverage per locus. *In silico* simulation is a valuable tool for any well‐designed RAD experiment. The R‐based package SimRAD (Lepais & Weir 2014) can be utilized for simulation‐based prediction of the expected number of loci for each RE (or their combination) and the genome of study. Although simulation estimates are likely to differ from the empirical data, valuable information can be gained to optimize experimental design before committing to the high cost associated with library construction and sequencing.

### Demultiplexing libraries

The files that are generated by the sequencer (typically FastQ files) require demultiplexing into individual samples based on nucleotide barcodes. The most popular packages for this task include *Stacks* (Catchen *et al*. 2011) and *pyRAD* (Eaton 2014). Standard quality control procedure is to discard sequence reads below user‐defined acceptable quality scores, erroneous barcodes and reads missing the characteristic sequence pattern obtained from the RE. Following demultiplexing, sequence files corresponding to each individual are generated for downstream analyses, including SNP calling and genotyping.

### SNP identification

One of the key advantages of RAD‐Seq approaches for nonmodel organisms (including many aquaculture species) is the ability to identify and genotype SNPs without requiring a reference genome for the organism under study. This approach, commonly defined in the literature as *de novo* assembly, can be performed using either *Stacks* (Catchen *et al*. 2011), *pyRAD* (Eaton 2014) or *dDocent* (Puritz *et al*. 2014b); however, the latter is limited to ddRAD or ezRAD data. The *de novo* approach involves identification and assembling of RAD loci in each individual, based on user‐defined parameters related to read coverage required per locus, and sequence divergence between loci (Catchen *et al*. 2011). Identification of SNPs and inference of alleles within RAD loci is performed using a maximum‐likelihood‐based algorithm (Hohenlohe *et al*. 2010), which undertakes statistical tests at each nucleotide position to assess the likelihood of a particular diploid genotype. In doing so, the model implicitly estimates and accounts for sequencing error rate (Catchen *et al*. 2011). The *Stacks* software does not currently support SNP identification and genotyping in the paired‐end (P2) read, unless anchored to a second RE (e.g. in ddRAD). Therefore, in original RAD experiments using Stacks, the P2 read is typically used for quality control (e.g. removal of PCR duplicates), and for constructing paired‐end ‘mini‐contigs’ which facilitate BLAST alignment and genotyping assay design (Etter *et al*. 2011). The simultaneous use of P1 and P2 reads in the case of *dDocent*, and the application of an alignment‐clustering algorithm in the case of *pyRAD*, allow the identification of insertion/deletion polymorphisms (indels) and identification of SNPs in the P2 reads.

Due to the decreasing cost of NGS, reference genome sequences are becoming available for many important aquaculture species. The number of species with reference genome assemblies is rapidly increasing (Atlantic cod, *Gadus morhua*, Star *et al*. 2011; Pacific oyster, Zhang *et al*. 2012; European sea bass, *Dicentrarchus labrax*, Tine *et al*. 2014; rainbow trout, Berthelot *et al*. 2014; Japanese eel, *Anguilla japonica*, Kai *et al*. 2014; half‐smooth tongue sole, *Cynoglossus semilaevis*, Chen *et al*. 2014; common carp, Xu *et al*. 2014b; Northern pike, *Esox lucius*, Rondeau *et al*. 2014; Nile tilapia, *Oreochromis niloticus*, Brawand *et al*. 2015; Asian sea bass, *Lates calcarifer*, Vij *et al*. 2016; Mediterranean mussel, *Mytilus galloprovincialis*, Murgarella *et al*. 2016; turbot, *Scophthalmus maximus*, Figueras *et al*. 2016; Atlantic salmon, Lien *et al*. 2016; channel catfish, Chen *et al*. 2016), and new sequencing data will improve genome quality and annotation. Therefore, reference‐guided RAD‐Seq approaches are likely to be increasingly utilized. Both *Stacks* and *dDocent* can utilize reference genome information, using standard alignment tools followed by similar SNP calling algorithms to the *de novo* approach described above.

### Potential bias and sources of error

While the bioinformatic pipelines for the RAD‐like approaches are becoming increasingly standardized, there remains potential intrinsic barriers that must be overcome to ensure the generation of accurate and repeatable SNP datasets. One example that is particularly relevant to the aquaculture research community is distinguishing between genuine allelic SNPs and paralogous variants resulting from ancestral whole genome duplication. This is particularly a challenge for salmonid species, and strategies to account for this include (i) assessing read coverage for patterns suggestive of paralogous variation, (ii) checking for excessive heterozygosity at loci and (iii) sequencing (double) haploid individuals as the basis for filtering out paralogous sequence variants (e.g. Everett & Seeb 2014; Houston *et al*. 2014; Palti *et al*. 2015a,b). Another potential source of error for all RAD‐Seq studies is the problem of RAD allele dropout (Gautier *et al*. 2013), where mutations within the recognition sequence for the RE segregating in the population are a common source of null alleles. The extent of the issue is related to the length of the RE recognition sequence, and it is therefore potentially more of a problem for ddRAD (which requires two REs) versus other methods (Gonen *et al*. 2015; Andrews *et al*. 2016). Both read coverage levels and assessment of segregation distortion in pedigreed crosses can assist in identifying and removing, or accounting for, these null alleles. Finally, the concept of PCR duplicates is raised above, and this is due to preferential amplification of certain clonal DNA fragments derived from the original genomic DNA fragments. PCR duplicates can give rise to the situation where one allele is overrepresented in the resulting sequence data and causes problems with differentiating homozygous and heterozygous individuals at that locus (Schweyen *et al*. 2014).

## Applications of RAD sequencing in aquaculture

Since its first description by Baird *et al*. (2008), RAD‐Seq has quickly spread through different fields of genetic research, and it has been used in different aquaculture species to construct genetic maps (e.g. Recknagel *et al*. 2013; Gonen *et al*.^2014^), for comparative genomics (e.g. Kakioka *et al*. 2013; Manousaki *et al*. 2015), for mapping genes associated with production traits (e.g. Houston *et al*. 2012; Shao *et al*. 2015; Fu *et al*. 2016), mapping sex determining loci (e.g. Palaiokostas *et al*. 2013a,b), studying population dynamics (e.g. Bradic *et al*. 2013), for fisheries management (e.g. Ogden *et al*. 2013), assembling reference genomes (e.g. Tine *et al*. 2014) or generating SNP resources for future SNP array development (e.g. Houston *et al*. 2014; Palti *et al*. 2014). A summary of the studies performed directly relevant for aquaculture is detailed below and in Table 2.

**Table 2:** Summary of aquaculture‐oriented studies using restriction‐site associated DNA sequencing (RAD‐Seq)

Study	Species	Aim	Technique	Samples	SNPs	Families
Salmonids						
Houston *et al*. (2012)	*Salmo salar*	Disease resistance QTL (IPNV)	RAD	32	6712	Two families
Gonen *et al*. (2014)	*Salmo salar*	Linkage map	RAD	96	8257	Two families
Campbell *et al*. (2014)	*Oncorhynchus mykiss*	Disease resistance QTL (BCWD and IHNV)	RAD	456	4661	40 families
Palti *et al*. (2014)	*Oncorhynchus mykiss*	SNP resource	RAD (×2)	19	145 168	19 genetic lines
Palti *et al*. (2015b)	*Oncorhynchus mykiss*	Disease resistance QTL (BCWD)	RAD	252	5612/4946	Two families
Liu *et al*. (2015b)	*Oncorhynchus mykiss*	Cortisol response to crowding QTL	RAD	234	4874	One family
Liu *et al*. (2015b)	*Oncorhynchus mykiss*	Disease resistance QTL (BCWD) and spleen size QTL	RAD	301	7849	Two half‐sib families
Vallejo *et al*. (2016)	*Oncorhynchus mykiss*	Genomic selection (BCWD)	RAD	711	24 465	81 families
Everett and Seeb (2014)	*Oncorhynchus tshawytscha*	Thermotolerance and growth QTL	RAD	422	3534	Six families
Larson *et al*. (2016)	*Oncorhynchus nerka*	Thermotolerance and growth QTL	RAD	491	11 457	Five families
Nonsalmonid fish						
Palaiokostas *et al*. (2013b)	*Oreochromis niloticus*	Sex determination QTL	RAD	88	3904/4477	Two families
Palaiokostas *et al*. (2015a)	*Oreochromis niloticus*	Sex determination QTL	ddRAD	372	1279	Five families
Palaiokostas *et al*. (2013a)	*Hippoglossus hippoglossus*	Sex determination QTL	RAD	93	7572/5954	2 half‐sib families
Palaiokostas *et al*. (2015b)	*Dicentrarchus labrax*	Sex determination QTL	RAD	187	6706	4 + 4 half‐sib families
Wang *et al*. (2015a,b)	*Scophthalmus maximus*	Sex determination and growth QTL	RAD	151	6647	One family
Brown *et al*. (2016)	*Polyprion oxygeneios*	Sex determination and growth QTL	ddRAD	59	1609	One family
Manousaki *et al*. (2015)	*Pagellus erythrinus*	Linkage map	ddRAD	99	920	One family
Shao *et al*. (2015)	*Paralichthys olivaceus*	Disease resistance QTL (*Vibrio anguillarum*)	RAD	218	13 362	One family
Palaiokostas *et al*. (2016)	*Sparus aurata*	Disease resistance genomic selection	2b‐RAD	777	12 085	75 families
Wang *et al*. (2015a,b)	*Lates calcarifer*	Growth QTL	ddRAD	144	3349	One family
Fu *et al*. (2016)	*Hypophthalmichthys nobilis*	Growth QTL	2b‐RAD	119	3323	One family
Invertebrates						
Jiao *et al*. (2014)	*Chlamys farreri*	Sex determination and growth QTL	2b‐RAD	98	7458	One family
Li and He (2014)	*Pinctada fucata*	Growth QTL	RAD	100	1381	One family
Shi *et al*. (2014)	*Pinctada fucata*	Growth QTL	2b‐RAD	98	10 577	One family
Tian *et al*. (2015)	*Apostichopus japonicas*	Growth QTL	2b‐RAD	102	11 306	One family
Lu *et al*. (2016)	*Marsupenaeus japonicus*	Thermotolerance and growth QTL	RAD	152	9829	One family
Dou *et al*. (2016)	*Patinopecten yessoensis*	Genomic selection (growth)	2b‐RAD	349	2364	Five families
Ren *et al*. (2016)	*Haliotis diversicolor*	Growth QTL	RAD	142	3317	One family

### Genetic marker discovery for SNP array development

Early studies using RAD‐Seq typically focussed on simply generating a genetic marker resource for nonmodel organisms. When the genome size of the target species is large, then whole genome (re)sequencing is arguably not cost‐effective for SNP discovery across many individuals, and genome complexity reduction is advantageous. As such, RAD‐Seq and similar techniques enabled a step change in the number of genetic markers (SNPs) available for several species (e.g. sturgeon, *Acipenser* genus, Ogden *et al*. 2013; or rainbow trout, Palti *et al*. 2014), and these have subsequently been used for several high‐resolution genetic studies. SNPs generated by RAD techniques have also been applied to produce SNP arrays for several aquaculture species, including Atlantic salmon (Houston *et al*. 2014), rainbow trout (Palti *et al*. 2015a) and Pacific oyster (Lapègue *et al*. 2014). With the reduction in sequencing costs over recent years, whole genome (re)sequencing (i.e. pool‐sequencing, Schlötterer *et al*. 2014) has become increasingly viable. However, RAD‐like techniques still hold a significant advantage for SNP discovery when (i) there is no reference genome available, and (ii) only a medium density SNP resource is required.

### Linkage maps and reference genome assembly

Restriction‐site associated DNA sequencing techniques have been widely used in aquaculture species for constructing genetic maps based on recombination events in defined crosses. Such medium density SNP linkage maps are useful tools for downstream applications such as QTL mapping, comparative genomic and gene mining, or population genomic studies. For example, RAD‐based linkage maps have been created for Atlantic salmon (Gonen *et al*. 2014), channel catfish (Li *et al*. 2014), Japanese flounder (Shao *et al*. 2015), turbot (Wang *et al*. 2015b) and Asian seabass (Wang *et al*. 2015a). Genetic maps based on RAD‐Seq have also contributed to mapping and orientation of scaffolds for reference genome assemblies for key aquaculture species such as European sea bass (Tine *et al*. 2014), rainbow trout (Berthelot *et al*. 2014), Japanese eel (Kai *et al*. 2014), half‐smooth tongue sole (Chen *et al*. 2014) and turbot (Figueras *et al*. 2016). While NGS technology has enabled rapid and cheap reference genome assemblies, they are typically fragmented and incomplete. Further, assembly errors are quite common, and linkage maps can also assist with resolving mis‐assemblies (Fierst 2015; Tsai *et al*. 2016a). Aquaculture species typically have an amenable family structure for high‐resolution linkage maps, due to the high fecundity resulting in large full and half sibling families. Linkage maps can also be used in conjunction with physical reference genome sequences to detect variation in recombination rates across the genome, with implications for downstream applications (e.g. LD between markers and QTL in association mapping studies).

### Mapping QTL associated with traits of economic importance

The rate of application of genomic technology to aquaculture species tends to reflect the degree of scientific and commercial interest of those species. This is typically motivated by the interest of understanding the genetic basis of economically‐important production traits, for example growth, disease resistance or sex determination. Researchers working in the high‐value salmonid species were amongst the first to exploit RAD‐Seq techniques, evaluating resistance to different pathogens causing high economic losses, including infectious pancreatic necrosis in Atlantic salmon (Houston *et al*. 2012), and infectious hematopoietic necrosis (Campbell *et al*. 2014) and bacterial cold water disease (Campbell *et al*. 2014; Liu *et al*. 2015a; Palti *et al*. 2015b) in rainbow trout. Based on early successes, and given the importance of disease resistance to modern aquaculture breeding programmes (Yáñez *et al*. 2014), large‐scale projects have been established to apply RAD‐like techniques to detect markers, and eventually the genes and causal mutations involved, for improving resistance. For example, the European Union funded FISHBOOST project (http://www.fishboost.eu) is using RAD sequencing techniques to genotype several thousand animals from large‐scale disease challenge experiments in rainbow trout, common carp, European sea bass, gilthead sea bream (*Sparus aurata*) and turbot. These genotype and phenotype data will be used to estimate genetic parameters, map disease resistance QTL and evaluate genomic prediction approaches for disease resistance breeding.

In addition to disease resistance, RAD‐Seq association studies have been widely applied for mapping QTL affecting a range of other production‐relevant traits, particularly in salmonid species. These include spleen size (Liu *et al*. 2015a) and cortisol response (Liu *et al*. 2015b) in rainbow trout, and thermal tolerance and growth in *Oncorhynchus nerka*, the sockeye salmon (Larson *et al*. 2016). Out with the salmonid genera, RAD‐Seq has been performed to map loci affecting disease resistance in olive flounder (*Paralychthys olivaceous*, Shao *et al*. 2015), and growth in bighead carp (*Hypophthalmichthys nobilis*, Fu *et al*. 2016) and turbot (Wang *et al*. 2015b). In addition, RAD‐like techniques have been very popular for marker discovery and QTL mapping in bivalve shellfish including Chinese scallop (*Argopecten irradians*; Jiao *et al*. 2014), Akoya pearl oyster (*Pinctata fucata*; Li & He 2014; Shi *et al*. 2014), variously coloured abalone (*Haliotis diversicolor*; Ren *et al*. 2016; Yesso scallop (*Patinopecten yessoensis*; Dou *et al*. 2016) and have also been applied in the shrimp kuruma prawn (*Marsupenaeus japonicas*; Lu *et al*. 2016) and one echinoderm, the sea cucumber (*Apostichopus japonicus*; Tian *et al*. 2015). Interestingly, 2b‐RAD has been the most common technique in bivalves, while in finfish, traditional RAD has been more widely utilized.

### Using RAD to study sex determination

Sex determination (SD) is one of the most critical traits for many aquaculture species, as phenotypic sex is often not evident in juveniles and sexual dimorphism in growth rate is commonly observed. SD is complex in many fish species, often with polygenic control and an environmental component (reviewed in Martínez *et al*. 2014), and the application of large genotyping projects has been strongly recommended to screen for SD loci in fish (e.g. Pan *et al*. 2016). RAD‐like techniques have clearly boosted our knowledge of SD in aquaculture, with studies in Nile tilapia (Palaiokostas *et al*. 2013a, 2015a), Atlantic halibut (*Hippoglossus hippoglossus*, Palaiokostas *et al*. 2013b), European sea bass (Palaiokostas *et al*. 2015b) and turbot (Wang *et al*. 2015b) finding putative sex determining loci. Controlling sex ratio is not only interesting to obtain higher growth rates, but also to avoid size dispersion or to delay sexual maturity. Further, there are some clear examples, like the sturgeon, where the commercial advantage of rearing fish of one sex over the other is obvious.

### Genomic selection approaches

While QTL mapping and MAS approaches can be successful when the genetic architecture of a trait suggests a gene of major effect (e.g. IPNV resistance, Houston *et al*. 2008; Moen *et al*. 2009), improvement of polygenic traits using genomic data is more effectively achieved using genomic prediction of breeding values (Meuwissen *et al*. 2001). Studies of genomic selection in aquaculture were first carried out in salmonid fish, with simulated (Sonesson & Meuwissen 2009; Lillehammer *et al*. 2013) and empirical (Ødegård *et al*. 2014; Tsai *et al*. 2015, 2016b; Vallejo *et al*. 2016) data, demonstrating the clear advantages over pedigree‐based methods. Studies using varying marker densities for prediction in salmonids have highlighted that as few as a thousand SNPs may be adequate for achieving the gain in selection accuracy versus pedigree approaches (Ødegård *et al*. 2014; Tsai *et al*. 2015, 2016b). Therefore, it is reasonable to assume that RAD‐like techniques may be useful for genomic selection in aquaculture breeding, as typical RAD SNP datasets comprise a few thousand SNPs. Indeed, the potential of this approach has already been highlighted for resistance to bacterial cold water disease in rainbow trout (Vallejo *et al*. 2016), for growth in Yesso scallop (Dou *et al*. 2016), and for resistance to pasteurellosis in gilthead sea bream (Palaiokostas *et al*. 2016).

### Genetic traceability and aquaculture sustainability

One of the main concerns for aquaculture producers and consumers is to minimize the environmental impact of fish farming. In this sense, traceability tools are essential to assess the impact of aquaculture escapees in natural populations or distinguish between farmed and wild specimens. RAD‐Seq has been utilized to obtain SNPs for sturgeon traceability and conservation (Ogden *et al*. 2013), which will contribute to enforce current legislation on aquaculture and fishing practices but also aid on the handling of wild stocks, critical for sustainable aquaculture. RAD‐Seq is also the main tool of the European project AquaTrace (aquatrace.eu), the results of which have been recently presented in the European Aquaculture Society meeting in Edinburgh (Aquaculture Europe 2016). One of the AquaTrace objectives was to assess the impact of escapees on natural populations of European sea bass, gilthead sea bream and turbot, while also developing forensically validated tools for traceability purposes. The results highlighted the utility of RAD‐Seq approaches to capture population or family specific variation making it a suitable tool for genetic traceability and conservation of natural populations. This is of the outmost importance for sustainable aquaculture growth, leading to lasting economic benefits, food safety and social acceptance.

## RAD‐Seq and SNP arrays, towards a peaceful co‐existence

The development of NGS has greatly increased the amount of genomic resources available in the most important aquaculture species, including genome assemblies for many of them. Alongside RNA‐Seq and whole genome sequencing, RAD‐Seq has contributed significantly to the availability of abundant genetic markers compared to a few years ago. While RAD‐Seq and similar techniques are likely to remain the genotyping method of choice for species with few genomic resources, several medium and high‐density SNP arrays are already available for aquaculture species (Atlantic salmon, Houston *et al*. 2014; Yáñez *et al*. 2016; channel catfish, Liu *et al*. 2014; common carp, Xu *et al*. 2014a; rainbow trout, Palti *et al*. 2015a; Pacific oyster and European flat oyster, Lapègue *et al*. 2014), and many more are unpublished or currently being produced and validated.

Single nucleotide polymorphism arrays are a type of DNA microarray, where hybridization of allele‐specific probes results in a fluorescent signal which can be measured to call a genotype in a given loci. They have both advantages and disadvantages over RAD‐Seq approaches (Table 3). For instance, the experimental procedures and bioinformatic analyses are much simpler for the user of SNP arrays, requiring less technical knowledge and usually resulting in a faster turnaround. The genotype scoring method is more robust and amenable to automation, and therefore less prone to errors (Hong *et al*. 2012; Wall *et al*. 2014). The repeatability and reproducibility are higher for SNP arrays than RAD‐Seq, and genotyped loci are known in advance. However, having a fixed set of loci on the chip is also a disadvantage, especially in species with strong population structure, because of ascertainment bias whereby the SNP set is biased to polymorphic markers in the discovery population(s). This presents a major issue where aquaculture strains for a specific species are highly variable, and the utility of a SNP array will vary hugely depending on the relationship to the discovery population. RAD‐like approaches overcome this issue and also offer much greater flexibility to the researcher in terms of the targeted number of loci. Further, RAD‐Seq captures variation that is specific to populations, families and individuals that is likely to be missed from SNP array, which are typically biased towards common variants. Another putative advantage of RAD‐like techniques is that the direct cost of the experiment is cheaper, although the additional time required for library preparation and bioinformatics analyses should be considered into any comparison.

**Table 3:** General comparison of restriction‐site associated DNA sequencing (RAD‐Seq) and single nucleotide polymorphism (SNP) chips

	RAD‐Seq	SNP arrays
Sample processing	Laborious	Straightforward
Bioinformatic analysis	Complex	Negligible
Turnaround time	Long	Medium
Accuracy	Medium‐high	High
Repeatability	Medium	High
Design	Adjustable	Fixed
Cost	Low	Medium

In the near future, genomic selection (GS) is likely to be a key technique for breeding programmes of many aquaculture species, due to the demonstrable increase in selection accuracy versus current pedigree‐based methods. SNP arrays are now routinely used in livestock breeding programmes for GS and are increasingly utilized in technologically advanced aquaculture breeding. Several studies have shown that only moderate SNP marker density is required for effective GS in salmon (Ødegård *et al*. 2014; Tsai *et al*. 2015, 2016b). Vallejo *et al*. (2016) compared both RAD‐Seq and SNP arrays for GS to BCWD resistance in rainbow trout, finding similar selection accuracies for both techniques despite higher marker density from the SNP chip (~40k SNP array versus ~10k RAD‐Seq). This may reflect high levels of linkage disequilibrium in typical aquaculture family selection programmes, whereby trait recording is often performed on close relatives of the selection candidates. Therefore, the higher marker density associated with SNP chips may be advantageous when predicting breeding values in animals more distantly related to the training population (Tsai *et al*. 2016b), or in species with greater effective population sizes and/or lower levels of linkage disequilibrium. However, given the relatively short genomes of many nonsalmonid aquaculture species (i.e. European sea bass – ~763 Mb, or turbot – ~658 Mb; Atlantic salmon – ~2970 Mb), the typical marker density generated by RAD‐like techniques may be perfectly adequate for effective GS. However, this needs to be tested, as the recombination frequency and patterns of linkage disequilibrium across the genome are pertinent to the question of adequate marker density. Further reductions in marker density requirements are likely to be observed when genotype imputation approaches are used, for example genotyping parents at high density, and offspring for a small subset of the markers. As already mentioned, RAD methods allow for substantial flexibility in terms of number of genotyped markers. In addition, lowering average sequence coverage in the offspring with parents sequenced at high coverage could be used to generate genotype data at a much lower cost.

## Targeted GBS techniques

Both RAD‐Seq and SNP arrays will also have to compete with recently developed genotyping methods based on targeted genotyping by sequencing. For example, genotyping‐in‐thousands by sequencing (GT‐Seq, Campbell *et al*. 2015) is a method of targeted sequencing which follows a multiplex PCR approach, where hundreds to thousands of loci (amplicons) are selected for genotyping. In this method, a multiplex PCR using loci‐specific primers that also contain Illumina sequencing primers is used to amplify the targeted regions. Unique barcodes for each sample are added with a second PCR reaction, followed by pooling and sequencing of samples. Unlike RAD techniques, this method requires previous knowledge to design the assays, and the number of SNPs genotyped in a single run is limited to a few thousand. Similar technologies are now provided by major genotyping technology providers, and it appears likely to become one of the most cost‐effective systems of genotyping targeted SNPs. Other GBS targeted‐sequencing techniques have also been recently developed, for example RAD capture (Rapture), where preselected RAD tags are isolated using capture probes and then sequenced (Ali *et al*. 2016). These targeted GBS techniques have the potential to become major players in aquaculture breeding and genetics due to their simplicity and flexibility. However, in part, they suffer from the same limitation as SNP arrays that they require prior knowledge and selection of the SNPs that are useful in the population of interest.

## Future outlook

Restriction‐site associated DNA sequencing techniques have driven a major increase in the application of genomics to aquaculture species. While the catalogue of SNP arrays for aquaculture species will increase in the coming years, it is likely that RAD techniques will continue to be widely applied. We anticipate that both techniques will co‐exist for several years, and the choice of RAD‐Seq or SNP chip will depend on the species and project‐specific factors. For example, it may be that high‐value aquaculture species with larger genomes (e.g. salmonids) are more suitable for SNP arrays, while lower‐value species with smaller genomes (and/or higher levels of LD) are more suitable for RAD techniques, although it will also depend on the resources available for each particular project. Targeted GBS techniques like GT‐Seq are likely to find a niche in genotyping hundreds to several thousands of previously identified SNPs across many samples. Further, RAD techniques are likely to remain the gold standard for new aquaculture species and/or those produced on a smaller scale, where SNP arrays are not available, and genomic resources are scarce. Eventually the cost of generating and analysing sequence data may drop to a level where genome complexity reduction is no longer required, but it seems unlikely in the short term. Therefore, RAD sequencing will continue to flourish in aquaculture research in the following years and is likely to be routinely applied to deliver the benefits of genomic selection to selective breeding of many different aquaculture species.

## Concluding remarks

The appearance of genotyping by sequencing technologies has provided the aquaculture research community with a hugely valuable method for identifying and concurrently genotyping large numbers of genetic markers in species with limited genomic resources. Further, these techniques have become multi‐purpose tools for addressing several topics of research and commercial interest like genetic diversity, population and family structure, association analyses with traits of economic interest, and genomic selection. Despite the increasing availability of genomic resources and the increasing number of SNP arrays, RAD techniques will continue being important for aquaculture research and application to selective breeding in the next few years. RAD sequencing and other genotyping by sequencing currently offer unequalled versatility and cost‐effectiveness for meeting the needs of many diverse research projects.
